# Anti-stigma advocacy for health professionals: a systematic review

**DOI:** 10.1080/09638237.2023.2182421

**Published:** 2023-03-15

**Authors:** Zoe Guerrero, Barbara Iruretagoyena, Sarah Parry, Claire Henderson

**Affiliations:** a Department of Public Mental Health, National Institute of Mental Health, Klecany, Czech Republic; b Department of Neurology and Psychiatry, Clínica Alemana Universidad del Desarrollo, Las Condes, Chile; c South London and Maudsley NHS Foundation Trust, London, UK; d Department of Health Service and Population Research, King’s College London Institute of Psychiatry, Psychology and Neuroscience, London, UK

**Keywords:** Anti-stigma, advocacy, healthcare professionals, systematic review

## Abstract

**Background:**

Many anti-stigma programs for healthcare workers already exist however there is less research on the effectiveness of training in skills for health professionals to counter stigma and its impacts on patients.

**Aims:**

The objective of this study was to examine the theory base, content, delivery, and outcomes of interventions for healthcare professionals which aim to equip them with knowledge and skills to aid patients to mitigate stigma and discrimination and their health impacts.

**Methods:**

Five electronic databases and grey literature were searched. Data were screened by two independent reviewers, conflicts were discussed. Quality appraisal was realized using the ICROMS tool. A narrative synthesis was carried out.

**Results:**

The final number of studies was 41. In terms of theory base, there are three strands - responsibility as part of the professional role, correction of wrongful practices, and collaboration with local communities. Content focusses either on specific groups experiencing health-related stigma or health advocacy in general.

**Conclusions:**

Findings suggest programs should link definitions of stigma to the role of the professional. They should be developed following a situational analysis and include people with lived experience. Training should use interactive delivery methods. Evaluation should include follow-up times that allow examination of behavioural change. PROSPERO, ID: CRD42020212527

## Introduction

In 1998, The Lancet published an essay by Norman Sartorius, entitled “Stigma: what can psychiatrists do about it?” (Sartorius, 1998). Focusing particularly on schizophrenia, he recommended that psychiatrists expand the focus of clinical work beyond symptom reduction to improving quality of life; reflect on and try to improve their own attitudes, by updating their clinical knowledge and learning from patients and their families about the impact of the illness and of stigma on them; monitor for discrimination and expand their role include to advocacy; and learn from others about how stigma and discrimination can be reduced.

More than twenty years later, it is worth considering the progress against these recommendations. Outcomes other than clinical ones are widely used in research and routine practice, and the concept of personal recovery has had a significant impact on mental health policy and practice in many countries (Le Boutillier et al., 2011). Further, continuing professional development is embedded in the requirements for license renewals and revalidation for many professionals. Reflective practice is used extensively in undergraduate and postgraduate training which in theory provides scope for examining one’s own attitudes to people with a mental disorder (Schutz, 2007). Stigma among health professionals, including mental health professionals, is an increasing focus of research (Henderson et al., 2014).

However, many people with mental disorders report contact with healthcare professionals as the most stigmatizing (Bates & Stickley, 2013). Health professionals’ stigma may lead to overlooking and underestimating the physical health of patients with mental health disorders (Liu et al., 2017). For example, people with mental health disorders receive a lower quality of care for physical health issues such as cardiovascular disease (Kugathasan et al., 2018) and diabetes (Mitchell et al., 2009), which may in part be due to lower referral rates of patients with mental health disorders to specialists or prescriptions (Corrigan et al., 2014). Moreover, health professionals often engage in stigmatizing practices such as labelling (Perry et al., 2020; Schulze, 2007), which may lead to not engaging with the patient or examining their problem fully (Carrara et al., 2019). These processes have been identified as contributors (Liu et al., 2017) to the reduced life expectancy in high-income countries of 15–20 years for people with severe mental illness, which may be even greater in low- and middle-income countries (Fekadu et al., 2015; Wahlbeck et al., 2011; Walker et al., 2015). It is useful to emphasise the power of health professionals as decision-makers at the individual and organisations levels, following Link and Phelan’s definition of stigma as the co-occurrence of labelling, stereotyping, separation, status loss, and discrimination in a context in which power is exercised (Link & Phelan, 2001).

Consequently, healthcare professionals have been the target of several national campaigns to reduce mental health-related stigma (One of Us in Denmark (Bratbo & Vedelsby, 2017), Opening minds in Canada (Stuart et al., 2014)). Trainings delivered through such campaigns have mainly focused on reducing provider bias and discrimination, showing short-term changes in knowledge and stigmatizing attitudes (Friedrich et al., 2013; Knaak, 2018). Reviews of anti-stigma interventions encompassing health professionals show similar findings and emphasise the need for longer term follow-up and the need to use behavioural outcome measures (Henderson et al., 2014; Mehta et al., 2015; Thornicroft et al., 2016). Their effect on providers’ behaviours and consequently patients’ health outcomes in the long term is unknown.

On the other hand, the potential for health professionals’ leadership in reducing the impact of mental health discrimination on their patients has not been examined extensively. Previous articles have acknowledged the potential impact that physicians’ advocacy could have in reducing discrimination (Arboleda-Flórez & Stuart, 2012; Thornicroft et al., 2010; Ungar et al., 2016), agreeing professionals could champion anti-stigma efforts, including much-needed structural changes as health care quality improvement and policy change work. The extent to which stigma reduces mental health professionals’ ability to provide effective care includes relative underfunding for mental health services, barriers to seeking and engaging with treatment, obstacles to rehabilitation due to discrimination in employment and within social networks, reluctance to pursue economic and social opportunities due to the anticipation of discrimination, and negative self-evaluation due to internalised stigma. However, there is little evidence that advocacy and effective stigma reduction methods have been incorporated into the role of psychiatrists or other mental health professionals, (Henderson et al., 2014; Mehta et al., 2015; Thornicroft et al., 2016; Zäske et al., 2014). As a result, how anti- stigma advocacy should be incorporated by mental health professionals is not yet clear.

The rationale for this review, therefore, is that we need to return to Sartorius’s recommendation and to learn from those fields of medicine in which there is an increasing focus on physicians’ social accountability and advocacy. Across North America, several organizations have expressed a pressing need for advocacy training in medical education (ACGME, 2007; Frank et al., 2015; Shaws et al., 2017). The Royal College of Physicians and Surgeons of Canada published a CanMEDS Physician Competency Framework, introducing health advocacy as one of six main competencies which medical education programs should address (Royal College of Physicians and Surgeons of Canada, 2011). The role of a health advocate is defined as to “identify and understand needs, speak on behalf of others when required, and support the mobilization of resources to effect change” (Frank et al., 2015). A series of articles in Canadian Family Physician (Buchman et al., 2016; Goel et al., 2016; Meili et al., 2016; Woollard et al., 2016) describes health advocacy activities in pursuit of social accountability at the levels of individual patients or families (micro), at the level of the local community (meso) and at the national or international level (macro). Training based on this spectrum, therefore, includes a variety of skills, whether this be helping a patient consider the pros and cons of self-disclosure of a concealable health condition (micro), working at the interface of health and other sectors to improve local services (meso) or campaigning for policy change (macro). As these may appear disparate, the ability to identify the right level of advocacy to address a particular issue necessitates such a spectrum approach. These skills can be taught at the undergraduate level to reinforce the idea of social accountability as part of professional identity, while postgraduate training can be tailored to specialty.

The aim of the present study is to examine the theory base, content, delivery, and evidence for the feasibility and effectiveness of interventions for healthcare professionals or healthcare students which aim to equip them with knowledge and skills to aid their patients to mitigate stigmatization and its health impacts. Such interventions may also aim to improve professionals’ or students’ attitudes to patients with stigmatised health conditions, particular individual characteristics, or from specific communities, but this cannot be the sole aim for inclusion in this review. To operationalise this aim, we address two research questions: “What are the theory base, content, and delivery methods of training for health professionals/students who provide direct patient care to reduce discrimination and its health impact on patients?” and “What is the evidence for the feasibility and effectiveness of programs with respect to knowledge, skills, and attitudes towards addressing discrimination and its health impact on patients?” Our future goal is for the results to inform the creation of interventions for mental health professionals, targeted at mental health stigma.

## Methods

We conducted a systematic review and narrative synthesis. A study protocol was written after initial scoping searches and background reading (available in PROSPERO, ID: CRD42020212527).

### Eligibility criteria

There were two sets of inclusion criteria, Table S1 in the supplementary material shows the PICO charts for both research questions. Participants’ inclusion criteria were common to both i.e. all had to be health professionals or healthcare students. Study design inclusion criteria differed: for the first research question any design was included while for the second research question empirical studies that reported feasibility outcomes e.g. recruitment and retention rates, satisfaction, or acceptability outcomes were included. All studies included had to be completed, therefore protocols were excluded. All languages were included, however, the abstract had to be in English.

**Table 1.** Data extraction.

Author year country	Target	Level	Rationale	Theory base	Contents	Hours	Methods	Trainers
Allen et al. (2013) Australia	Nursing students	Interpersonal	Nurses are not able to directly overcome many structural barriers to health, such as poverty and low socio-economic status. However, nurses are able to provide culturally appropriate anti-discriminatory health care and understand the complex effects of the social determinants of health	Social constructivist model of health, (Baum, 2011) theory of transcultural nursing (Leininger & McFarland, 2002)	Knowledge on stigma and social conditionings of health, anti-stigma project, advocacy skills, direct contact (clinical placement).	72 h	Self-reflection, class discussion, small group discussion, case-scenarios, written assignment, clinical placement.	Faculty
Bakshi et al. (2015), USA	Medical students	Structural	Prioritizes engagement with local underserved communities, focusing on collaborations with community-based organizations Aims to equip the next generation of physicians with the ideals, peer support, knowledge, and skills to help eliminate health inequities through systems-level change	Social justice as defined by American Board of Internal Medicine	Human rights knowledge, condition knowledge, social justice knowledge, advocacy skills, career development knowledge, research skills.	8 weeks	Interactive lecture, class discussion, mentorship, personal projects (research and advocacy), practical experience (clinical rotation)	Faculty, expert speakers, older students
Boutain (2008) USA	Nursing students	Structural	The commitment to social justice is core in the nursing field There is a lack of evaluation of social justice in practice – there is a need for a tool to do so	Social justice	Social justice knowledge, advocacy skills, direct contact.	Not reported	Interactive lecture, essay writing, practical experience, self-reflection	Faculty
Burdett et al. (2010) UK	Nursing students	Interpersonal	Not reported	“Old people champion” program	Knowledge on stigma. Direct contact (speaker), indirect contact (video), advocacy skills, anti-stigma interventions.	6 hours	Simulation, case- scenario, small group discussions, poster presentation, video.	Trained faculty
O’Carroll and O’Reilly (2019) UK	GP	Interpersonal	It has been established that GPs who work with deprived or marginalised populations require context-specific knowledge and skills appropriate to the morbidity and mortality profile of those populations and that current medical education programmes fail to address these educational needs	Allport′s contact theory (Allport, 1954)	Knowledge on social determinants of health and trauma informed care. Self- care skills, direct contact (clinical placement), identify own bias skill, advocacy skills, recognize stigma.	128 h	Clinical placement, self- reflection, interactive lectures, theatre workshop, class discussion.	North Dublin GP training programme staff
Crawford et al. (2017) Australia	Occupational therapy students	Interpersonal, Structural	Client groups seen by occupational therapists do not have their rights fulfilled Occupational therapists require knowledge and confidence regarding human rights if they are to work effectively Occupational therapists can, and should, drive social change	Human rights (Hocking et al., 2021)	Human rights knowledge, Communication skills (including social media), cultural competence knowledge, advocacy skills, personal project.	13 wk	Interactive lecture, self-reflection, case-scenarios, anti-stigma project.	Faculty
DallaPiazza et al. (2018) USA	Medical students	Interpersonal	Teaching medical professionals about structural racism and how to recognize and address bias in clinical encounters has become increasingly imperative.	Jones Tripartite Model of Racism (Jones, 2000)	Knowledge on stigma, social determinants of health and trauma-informed care. Skills to address stigma, mnemonic frameworks to address personal unconscious bias and external microaggressions	11 h	Interactive lecture, small group case-based discussion, self-reflection, brainstorm, video	Faculty, student peers guided discussions
Delashmutt and Rankin (2005) USA	Nursing students	Interpersonal, structural	To be effective care givers, nurses must have an understanding of the complex nature of poverty, especially the multifaceted health challenges faced by the poor.	Empowerment	Knowledge on stigma, human rights and legislation. Direct (clinical placement, speaker), indirect contact (video) identify self-bias skill, advocacy skills.	32 h	Class discussion, interactive lectures, self-reflection, written assignment, video, clinical placement.	Faculty
Dharamsi et al. (2010) Canada	Medical students	Structural	Students should respond to the broader determinants of health by linking core concepts from the humanities and social sciences with the clinical and basic sciences. Help students appreciate first-hand the impact of social determinants on health outcomes	Canadian Medical Education Directions for Specialists (CanMEDS) health advocate role	Social determinants of health, applied advocacy skills	8 wk	Practical experience (over-seas placement), personal project, essay writing, self-reflection (reflective journal)	Faculty and clinical staff
Ezedinachi et al. (2002) Nigeria	Health workers at general hospital	Interpersonal	Health professionals should take the lead in the protection of human rights Addresses issues of stigmatization and human rights as integral to appropriate care of people with HIV disease, and the link between stigma and HIV	Not reported	HIV information, stigma knowledge, human rights knowledge.	2 d	Interactive lectures, role plays, small group discussion, group discussions, videos.	Experts, influential role models trained in initial workshops.
Fisher et al. (2017) USA	Social workers	Interpersonal	Not reported	Biopsychosocial factors under racism. Interracial interaction (Kratzke & Bertolo, 2013; Sue et al., 2009) microaggressions (Boysen, 2012)	Knowledge on stigma and biopsychosocial model of health. Anti- stigma skill.	3 h	Small group discussion, class discussion, role play, brainstorming	Multiracial facilitators
Fisher-Borne (2009) USA	Health care professional (Disease intervention specialist)	Interpersonal	A key strategy in addressing health disparities is promoting cultural competency among health professionals.	Theory of cultural humility, Campinha-Bacote model of cultural competence (Campinha-Bacote, 1999)	Knowledge on race, MSM/LGBT, cultural competence and stigma. Identify own bias, advocacy skills, communication skills, indirect contact (video), direct contact (speaker). Resource manual with LGBT specific local and state-wide resources	16 h	Role playing, case-scenarios, brainstorm, self-reflection, dynamic, paired-sharing.	Project STYLE staff
Flatt-Fultz and Phillips (2012) USA	Healthcare professional working with people with intellectual disabilities	Interpersonal	Empowerment is the first important step for agencies that support people with intellectual and developmental disabilities. Training focused explicitly on empowerment was not investigated before	Cattaneo and Chapman empowerment (Cattaneo & Chapman, 2010)	Indirect contact (video)	0.5 h	Video, group discussion.	Not reported
Geibel et al. (2017) Bangladesh	Healthcare professional working in sexual and reproductive health and rights	Interpersonal	Working with health providers to reduce stigma and discrimination in the healthcare setting is one strategy to improve service utilization and quality of care	Not reported	Knowledge on stigma, human rights and HIV. Advocacy skills at an interpersonal and structural level.	24 h	Interactive lecture, group discussion, self-reflection, use of pictures, game, role-play, personal project.	Specialized trainers (Link up project)
Gonzalez et al (2015) USA	Medical students	Interpersonal, Structural	Curricula which instructs students on reducing health disparities both within clinical practice and within their communities. Promote medical students’ awareness of their own potential to contribute to health disparities and to provide them with clinical and advocacy skills to reduce systemic causes of health disparities.	Not reported	Anti-stigma project, advocacy skills, health disparities knowledge, social determinants of health knowledge, implicit bias knowledge (IAT test), communication skills, Advocacy skills (strategic planning, grassroots organizing, meeting with legislators, and media communications)	19.5 h	Interactive lectures, group discussions, role play, small group discussion, case scenarios, personal project.	Faculty
Gonzalez et al. (2020) USA	Medical students	Interpersonal	Curricula usually emphasize increase awareness of implicit bias but don’t provide opportunities for skill development and practice.	Conceptual framework by Teal and colleagues explaining individual’s progression through various stages related to IBRM (Teal et al., 2010)Transformative learning theory.	Implicit bias knowledge, implicit bias recognition skills, communication skills, advocacy skills, address biased comments skills in clinical and teaching encounters, skills to manage own bias.	13.5 h	Interactive lecture, contact, videos of popular culture, write personal narratives, self-reflection on students own implicit bias and lived experience, race implicit association test, debrief, case-scenarios, recorded role-play with “do-over”, brainstorm.	Not reported
Griffith and Kohrt (2016) USA	Psychiatry residents	Interpersonal	Not reported	Social psychology and social neuroscience	Knowledge on stigma and recovery. Anti-stigma project, skill aid coping with stigma skill, framework for anti-stigma strategies.	12 h	Small group discussion, interactive lectures, case- scenarios, role-play, readings, games	Faculty
İnan et al. (2018) Turkey	Nursing students	Interpersonal	Nurses who are in contact with patients, their relatives, and other members of society have the opportunity to be role models for creating positive attitudes.	Not reported	Knowledge on mental illness and stigma. Direct contact (placement, visit) and indirect contact (video). Anti-stigma campaign. Skills on communication and aiding in coping with stigma.	32 h	Group discussion, interactive lectures, personal project, videos, article review. Clinical placement and observership	Faculty and stigma expert
Jindal et al. (2022) USA	Pediatric residents	Interpersonal, structural	Medical education should build provider knowledge and capacity to address racism.	Not reported	Self-reflection and implicit bias, historical trauma, structural racism,	1 h	Interactive lecture, group discussion, role-playing	Faculty
Jones and Smith (2014) USA	Nursing students	Structural	The American Association of Colleges of Nursing (2008) states that advocacy “is a fundamental aspect of nursing practice”. Preparing nurses for leadership in areas of advocacy and policy development allows them to develop a vision for how nurses contribute to creating healthy communities.	Not reported	Knowledge on health disparities, human rights, social justice and marginalized populations. Advocacy skills. Assignment on advocate for current issue affecting vulnerable population (policy statement). Indirect contact (video)	Nor reported	Group discussion, interactive lecture, class presentation of assignment, video	Faculty
Knaak (2018) Canada	Nurses in general hospitals	Interpersonal, Structural	Healthcare professionals can be stigmatizing Healthcare professionals report that they lack the skills to help someone with a mental health issue, which also contributes to stigma.	Not reported	Stigma knowledge, strategies to address stigma, recovery model knowledge, social determinants of health knowledge, advocacy skills.	Not reported	Self-reflection, case-scenarios, web-based.	Not reported
Lax et al. (2019) USA	Residents	Interpersonal, Structural	Needs assessment showed that healthcare professionals do not know how to address social determinants of health Pilot study: Implementation of an advocacy teaching- module enabled residents to screen for and document social determinants of health consistently	Health advocacy (Wright et al., 2005)	Advocacy skills, framework, community resources, legislative knowledge.	Not reported	Case-scenarios, interactive lectures, class discussion, case-scenarios, interactive lectures, small group discussion, practical experience.	Faculty-resident pair. Experts speaker guests.
Li et al. (2013) China	Health care professional in general hospital	Interpersonal, structural	Stigma in the general population has been well‐documented, but its impact is also felt in healthcare settings, where it can lead to testing avoidance, barriers to health counselling and a lack of adherence to antiretroviral therapies.	Not reported	Knowledge on HIV and stigma, indirect contact (video), communication skills, advocacy skills, identify bias.	10.5 h	Group discussion, games, role- playing, pair-sharing, demonstrating, case- scenarios, video.	Trained local health educators, AIDS specialists, project staff
Li et al. (2015) China	Community mental health staff	Interpersonal	Interventions are needed to address the stigma of mental healthcare professionals	Not reported	Condition knowledge, public health knowledge, stigma knowledge, legislative knowledge, rehabilitation knowledge.	14 d	Clinical practice, interactive lectures.	Not reported
Mason and Miller (2006) USA	Social work students	Interpersonal	Students need an in-depth under- standing of mental health disorders so they may provide balanced and focused treatment for each client’s unique abilities and growth potential.	Not reported	Knowledge on stigma. Indirect contact (video), direct contact (clinical placement in group and individual sessions targeting auto-stigma with patients), skills to aid coping with stigma, recognize own bias, social isolation.	Not reported	Interactive lecture, role play, group discussion, educational videos, clinical placement.	Faculty
McAllister (2008) Australia	Nursing students	Interpersonal	Clinicians need cultural and social skills to influence public opinion, enhance tolerance, deepen understanding, explode myths, and work to effect social change. critical literacy is an important cognitive skill, effective in raising consciousness about inequity and injustice.	Critical literacy	Knowledge on mental health, social justice, and stigma. Skills on advocacy and critical literacy, indirect contact (video), framework for critical literacy	120 h	Interactive lectures, readings, video, textual analysis of different sources (musical representations, film, news).	Faculty
Nelson et al. (2015) USA	Medical residents	interpersonal	Physicians receive little to no training on the topic of race and racism, yet this is one of the largest barriers to health equity.	Not reported	Knowledge on stigma. Advocacy skills, identify own bias skill. Framework ("interrupt and educate") for addressing racism, commitment to address racism.	6 h	Group discussion, interactive lectures, video.	Faculty
Potts et al. (2022) UK	Medical residents	Interpersonal	Healthcare professionals should be a main target of anti-stigma campaigns as they can be highly stigmatizing towards their patients, however healthcare professionals could also be those who aid anti-stigma efforts	Recovery oriented	Knowledge on stigma, anti-stigma skills, direct (speaker)	2.5 h	Interactive lecture, group discussion, video	Psychiatrist, people with lived experience
Üstün and İnan (2018) Turkey	Nursing students	Interpersonal, systemic	Knowledge transmission on stigma is not enough, there is a need to use different educational methods to raise awareness and impart change.	Not reported	Anti-stigma project, stigma knowledge, identify own bias skills.	16 h	Personal project, class discussion, video, interactive lectures, case-scenario, brainstorm	Not reported
Shah et al. (2014) India	Nursing students	Interpersonal	Across multiple studies, stigma toward PLHIV has been found to be high among health care workers, including nurses and ward attendant	Not reported	Knowledge on HIV and stigma. Direct contact (speaker), strategies to decrease stigmatizing behaviours in hospital.	2 h	Interactive lecture, brainstorm, group discussion.	4th year medical student, PLHA
Sheely-Moore and Kooyman (2011) USA	Mental health students	Interpersonal, structural	It is imperative for counsellor educators and trainers of mental health professionals to infuse instructional strategies that promote multicultural and social justice competencies for trainees.	Multicultural competences (Arredondo et al., 1996). Lee’s (2018) model of self- exploration for social justice.	Knowledge on stigma, anti-stigma project, direct contact (visits), advocacy skills, identify own stigma.	Not reported	Class discussion, self- reflection, modelling, case scenarios, role play, simulation, videos, obervership.	Faculty
Sherman et al. (2019) USA	Family medicine residents	Interpersonal, structural	There is a need for a health professionals curriculum that will move beyond simply identifying implicit biases through self-reflection to (a) provide insight into how such insidious biases perpetuate institutional inequities and potentially exacerbate structural racism, and (b) empower health care professionals with skills for managing instances of racism and other implicit biases in their professional lives.	Not reported	Knowledge on race-culture, stigma and implicit bias. Emotion regulation skills, commitment, self bias recognition skill, tools to address barriers for health care.	1.5 h	Interactive lectures, group discussion, self-reflection, video.	National experts on implicit bias
Sukhera et al. (2020) Canada	Health professionals	Interpersonal	Traditional stigma reduction education programs often fail to confront the implicit nature of stigmatizing attitudes.	Implicit bias	Knowledge on stigma. Communication skills, identify own bias skills, “facilitating awareness through dissonance” dynamic.	4 h	Interactive lecture, group discussion, small group discussion, role play, case-scenario, self-reflection, debrief	Physician and nurse facilitator
Tucker et al. (2020) USA	Medicine students	Interpersonal	It is key to understand the impact of mental illness on individuals and their families and increase patient-centered and collaborative care in emergency and routine practice settings	Not reported	Understanding the experience of mental illness, supporting predictable emotions and needs, empathy’s role in effective treatment, psychological elements of collaborative care, and applying collaborative treatment principles	15 h	Interactive lecture	It is led by a team of three trained facilitators, as follows: (1) a person living well in recovery from SMI, (2) a family member of someone with SMI, and (3) a healthcare provider with personal or family experience with SMI.
Uys et al. (2009) Lesotho, Malawi, S. Africa, Swaziland Tanzania	Nurses	Interpersonal	Not reported	Empowerment by Cross and Choudhary (Cross & Choudhary, 2005)	Knowledge on stigma, anti-stigma project, direct contact (develop project together), aid coping with stigma skills, advocacy skills	24 h	Group discussion, interactive lectures, person project	Nurse, PWLA
Wagaman et al. (2019) USA	Social work students	Interpersonal, structural	Transformational models of teaching and learning are needed to explore and expand the preparation of students to address race and racism in practice	Critical race theory (Abrams & Moio, 2009), liberation theory (Brigham, 1977)	Knowledge on stigma and social justice. Recognize own bias, direct contact (community visit), advocacy skill, commitment, resources to extend work in community.	8 h	Interactive lecture, small group discussion, brainstorm, self-reflection, group discussion, observership.	Community worker
Webb and Sergison, (2003) UK	Health care and social worker in child health services	Interpersonal	Not reported	Not reported	Knowledge on race and stigma. Empathy skills, communication skills, indirect contact (video).	8 h	Case scenarios, videos, interactive lectures, group discussion.	Multidisciplinary group, experts in antidiscrimination
Werkmeister Rozas and Garran (2016) USA	Social work students	Interpersonal, structural	Recently, in the USA, there have been more concerted efforts to infuse human rights throughout social work curricula and, as a result, there is a call for ways to evaluate and/or measure student outcomes	Not reported	Knowledge on stigma, advocacy skills, anti-racism project, identify stigma skills	Not reported	Interactive lectures, class discussion	Faculty
White-Davis et al. (2018) USA	Healthcare professionals	Interpersonal	While teaching health care professionals about racism reduces biases, few curricula. for medical education exist.	Jones Tripartite Model of Racism (Jones, 2000)	Knowledge on stigma. Anti-racism commitment. Toolkit for exploring racism.	1.5 h	Interactive lecture, small group discussion, case-scenario, group discussion, video	Multicultural health faculty (multiracial, multidisciplinary team)
Wu et al. (2019) USA	Healthcare professionals of various settings	Interpersonal structural	Even if individual health care professionals are knowledgeable, institutional support is needed to facilitate constructive discourse and enact broad change. Without foundational knowledge and institutional policies, trainees and providers can be subject to a “silent curriculum” of the health care system, in which biased behaviours and values are internalized and perpetuated	Anti-oppressive practice (Larson, 2008) social justice (Sensoy & DiAngelo, 2012) Jones Tripartite Model of Racism (Jones, 2000)	Stigma knowledge, advocacy skills, identify own bias skill, compassion, emotion regulation skill, anti-racism commitment. Step up/step back framework for addressing racism.	3 h	Small group discussion, interactive lectures, group discussion, brainstorm, case- scenarios, self-reflection, interactive activities	2 authors
Zäske et al. (2014) Germany	Mental health providers at psychiatric hospital	Interpersonal	There is a strong power dynamic between psychiatric staff and patients. Which may lead to high levels of institutional stigma. It is important to work with psychiatric staff as they can become role models both within and outside of their institution when it comes to detecting and addressing stigma	Not reported	Knowledge on stigma and professional role, direct contact (speaker), anti-stigma skills.	16 h	Role playing, group discussion, interactive lectures, short- essay writing.	Trainer experienced in education and people with lived experience

### Information sources and search strategy

Data were collected through five different databases: MEDLINE (via Ovid) to search studies from the medical environment; PsycINFO (via Ovid) to search studies from the mental health stigma perspective, CINAHL (via EBSCO) for healthcare and nursing-specific articles; EMBASE to cover more fields of healthcare; and ERIC (via EBSCO) for literature on further education. Grey literature was also hand searched via the meta-search engine DogPile. The date last searched for the above databases was the 8th of May 2022. The terms used in the search were classified into four themes, health professionals terms such as “Health$professional” or “nurs$”, stigma terms such as “stigma” or “discrimination”, content terms such as “health advocacy” or “social accountability” and delivery terms such as “training” or “curriculum”. MeSH terms were also used for the appropriate databases. Titles and abstracts from 1980 and onwards were searched. The search strategy used was:

“Health professional terms” tw AND (“Intervention terms” adj3 “Stigma terms”) ti

OR

((“Culturally competent care” OR “Cultural competenc*”) adj3 “intervention terms”) ti AND “Health professional terms” tw AND “Stigma terms” tw

OR

(Health professional MESH terms AND Stigma MESH terms AND Intervention MESH terms)

All the search terms used can be found in the supplementary data Table S2.

**Table 2.** Efficacy outcomes

Reported by target population	Zaske et al.	Inan et al.	White-Davis et al.	Potts et al.	Li et al.	Wu et al.	Fisher-Borne et al.	Allen et al.	Nelson et al.	Uys et al.	Shah et al.	Geibel et al.	Sukhera et al.	Li et al.	Lax et al.	Knaak et al.	Gonzalez et al.	Ezedinachi et al.	Tucker et al.	
**Attitudes**																				
Social distance	↙	↙																	↙	
Commitment to personal change			↑																	
Attitude towards condition/characteristic				↑			→	→		→		↑	↑	↑*	→	↑	↑	↑		
Endorsement of coercive policies											↑									
Willingness to provide care												↑ *								
**Knowledge**																				
Knowledge of stigma			↑	↑		↑	↑		↑					↑^			↑			
Policies against discrimination												↑								
**Self-confidence**																				
Confidence in practicing trans-cultural nursing								↑												
Confidence in addressing discrimination			↑												↑		↑		↑	
**Behaviour**																				
Discussing stigma	↑				↑						↑									
Discriminate when dispensing medication																				
Discriminate when drawing blood											→									
See PLHIV as a client												↑								
General reported and intended behaviour														↑^						
Advocating self-reported practices															↑					
**Skills**																				
Ability to deliver care				↑			→		↑									↑		
Identify discrimination	↑					↑												↑		
Address discrimination	↑		↑			↑	→													
Managing prejudice situations	→					↑														
**Reported by observers**																				
Perceived stigma reduction					↑															
Improvement in equal treatment					↑															
**Reported by stigmatized population**																				
Perceived stigma										↑		↑								
Satisfaction with quality of care										↑		↑								
Self-stem										↑										
Self-efficacy										→										
Report discussing stigma with provider												↑*								

*Key*: * = at 6 mo. ^ = at 6 and 12 mo. ↑ increase → constant ↙ decrease

### Study selection

In the first stage of screening for abstract and title, two co-reviewers (ZG, BI) screened all of the data independently then discussed conflicts until they reached a mutual agreement. In case of persistent conflict, a third reviewer was consulted (CH). In the second stage of full-text screening, the data were divided in two halves which each co-reviewer (ZG, BI) screened independently. Before screening all data the same two co-reviewers screened 10% of each other’s data set in order to achieve mutual agreement. Conflicts were discussed until mutual agreement was reached, if conflict persisted a third reviewer was consulted (CH).

For the data extraction stage data remained split between two co-reviewers as it was in the full-text screening stage. Data extraction was firstly done for 10% of the data by the same two independent co-reviewers, conflicts were discussed, following which co-reviewers extracted data for their half of the data each.

### Data extraction

A bespoke data extraction tool was used. Included studies were classified according to the authors, origin, year of publication, sample characteristics, methods, and main results. For research question 1, data on the theory base, content, and delivery of the programs were collected while, for research question 2, data on effectiveness measures and their outcomes were collected. Outcome measures were categorised based on a definition of stigma as a problem which arises from a lack of knowledge such as ignorance and misinformation, negative attitudes fuelled by prejudices, and excluding or avoiding behaviours, otherwise called discrimination (Thornicroft, 2008).

### Data synthesis

The data were analysed and synthesized using narrative synthesis (Popay et al., 2006). The data were categorized and synthesized based on: the research questions they pertain to; the type of discrimination they address, this was defined via the rationale of each study and the theory base which they employed for example social justice models or human rights models; and finally, the level at which they destigmatize – individual or structural.

### Quality appraisal

Quality appraisal was carried out using the ICROMS (Integrated quality Criteria for the Review of Multiple Study designs) tool, however, studies which scored low were not excluded. This was done independently by two co-reviewers on 10% of the papers. This tool was chosen as it is one of the few tools which allows to assess studies of different designs. The ICROMS tool uses a list of seven quality criteria (aims and justifications, sampling bias, bias in measurements and binding, bias in follow-up, bias in other study aspects, analytical rigour, and bias in reporting/ethical considerations). Each criterion is scored according to a scoring matrix which describes the lowest possible score each study design should achieve for inclusion (Zingg et al., 2016).

## Results

**Figure 1.**
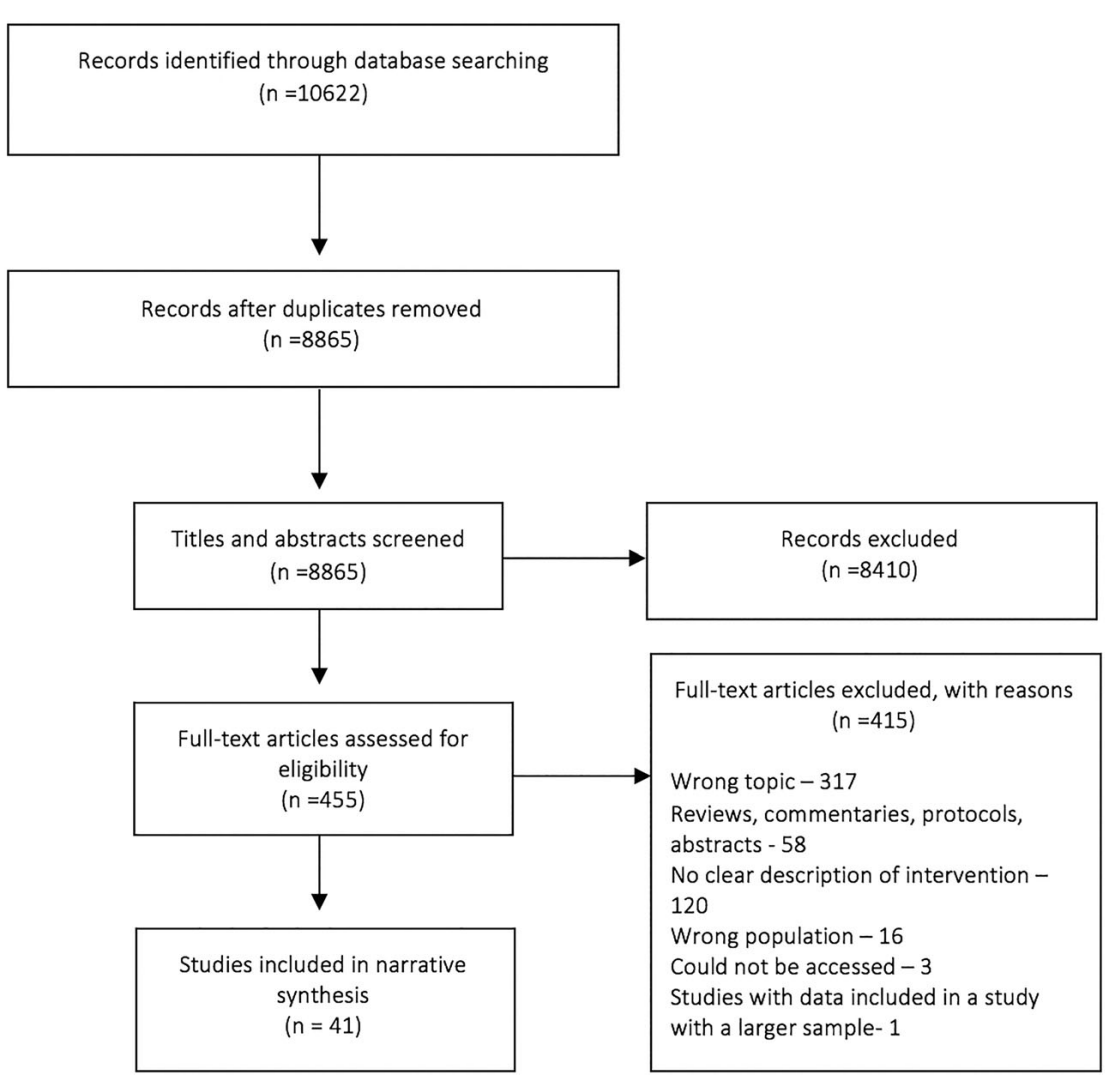
PRISMA flow diagram.

The total number of papers identified was 10,622, and 8865 after removing duplicates, one paper was identified by one of the review authors (CH) as she is the study principal investigator. After the first stage of screening for abstracts and titles, 453 papers were included. After inspection of the full texts of the 455 papers 41 were included for final data extraction. Also, three other papers were not processed in the data extraction stage as they are protocols or descriptive studies but were described as they are of interest for future reference. Most papers excluded at the full-text stage were ineligible because the programs they described were either focusing solely on destigmatizing healthcare professionals and not training them to help their patients, or solely measured attitudes in healthcare professionals. The exclusion reasoning can be seen in the PRISMA flow chart below (Moher et al., 2009) (Figure 1).

The data extracted can be seen in Table 1. Most of the studies were done in high-income countries, most in the USA or Canada (Bakshi et al., 2015; Boutain, 2008; Crawford et al., 2017; Dharamsi et al., 2010; Flatt-Fultz & Phillips, 2012; Gonzalez et al., 2015, 2020; Jindal et al., 2022; Knaak, 2018; Lax et al., 2019; Sukhera et al., 2020; Tucker et al., 2020; White-Davis et al., 2018; Zäske et al., 2014). Four studies included data from middle and low-income countries (Ezedinachi et al., 2002; Geibel et al., 2017; Potts et al., 2022; Uys et al., 2009) In terms of participants, 24 studies focused on healthcare students, be that nursing or medicine, describing programs added to teaching curriculums at universities (Allen et al., 2013; Bakshi et al., 2015; Boutain, 2008; Burdett et al., 2010; Crawford et al., 2017; DallaPiazza et al., 2018; DeLashmutt & Rankin, 2005; Dharamsi et al., 2010; Gonzalez et al., 2015, 2020; İnan et al., 2019; Jones, 2000; Jindal et al., 2022; Lax et al., 2019; Mason & Miller, 2006; McAllister, 2008; O Carroll & O’Reilly, 2019; Potts et al., 2022; Shah et al., 2014; Sheely-Moore & Kooyman, 2011; Tucker et al., 2020; Üstün & İnan, 2018; Wagaman et al., 2019; Werkmeister Rozas & Garran, 2016). A small proportion of studies focused on participants with work experience, such as therapists or specialist mental healthcare staff (Ezedinachi et al., 2002; Fisher et al., 2017; Fisher-Borne, 2009; Flatt-Fultz & Phillips, 2012; Knaak, 2018; Li et al., 2015, 2013; Nelson et al., 2015; Sherman et al., 2019; Sukhera et al., 2020; White-Davis et al., 2018; Zäske et al., 2014).

### Quality appraisal

Twelve studies could not be assessed because they did not have an empirical study design. Of the remaining 29, the ICROMS minimum scores were achieved for 14 of the 29 studies. The studies which did not achieve the minimum scores lacked enough information about participant recruitment and sampling, blinding, prevention of detection bias or contamination between groups. See Table S3 in the supplementary material for all ICROMS scores.

**Table 3.** Acceptability outcomes.

Author year country	Design	Sample size	Sampling	Nature of intervention	Timing of outcome measurement	Acceptability outcome	Response rate
Burdett et al. (2010) UK	Cross-sectional	325	Convenience	Mandatory academic activity	Post-intervention	**•**70% rated the session excellent. **•**56% thought the structure was excellent, 42% thought it was good. **•**66% rated content as excellent, 33% as good. **•**100% would recommend the session.	Not reported
DallaPiazza et al. (2018) US	Cross-sectional	536	Convenience	Mandatory academic activity	Post-intervention	**•**63% agreed to a great and 26% to a moderate degree that they felt more comfortable using concrete tools delivered. **•**71% agreed to a great and 21% to a moderate degree that additional training on this topic would be beneficial. **•**72% felt objectives had been met considerable/in very high degree.	30–41%
Knaak (2018) Canada	UPPI	198	Self-selected convenience	Voluntary	Post-intervention	**•**87.6% strongly agreed or agreed the program addresses their learning needs. **•**93.8% strongly agreed or agreed it was relevant to their practice. **•**92.2% strongly agreed or agreed it enhanced their knowledge in the area. **•**78.1% strongly agreed or agreed they would recommend it.	77.16%
Shah et al. (2014) India	CPPI	91	Convenience	Mandatory academic activity	Post-intervention	**•**89–100% rated it as useful and easy to understand. **•**95% felt it will change their practice and would recommend it. **•**9–22% reported some of the materials made them uncomfortable. **•**38–40% report feeling embarrassed to share their opinions. **•**87–93% felt they could be honest about their beliefs.	100%
Sherman et al. (2019) US	Cross-sectional	31	Not reported	Not mentioned	Post-intervention	**•**88% qualified it as excellent and 13% as very good. **•**100% would strongly recommend it.	100%
Webb and Sergison, (2003) UK	Cross-sectional	140	Self-selected convenience	Voluntary	Huddersfield: Post-intervention Cardiff: 2–7 years post-intervention	Huddersfield: **•**98% rated the course as good or excellent. **•**75% said training met most to all of their needs. **•**99% stated the course outcomes were achieved. **•**96% were satisfied/very satisfied with the practical info given. **•**21% said they would like further training Cardiff: **•**83% rated the course as relevant or highly relevant. **•**79% said this was the first such training attended. **•**52% felt they would like further training.	Huddersfield: 97%, Cardiff: 62%

CPPI: controlled pre-post study.

### Research question 1 – What are the theory base, content and delivery methods of the programs?

#### Theory base

Fourteen of the 41 studies included considered the idea that the goals of the program should align with the vision of the curriculum or the role of the professional themselves (Bakshi et al., 2015; Boutain, 2008; Dharamsi et al., 2010; Geibel et al., 2017; Gonzalez et al., 2015; Jones, 2000; Jindal et al., 2022; Lax et al., 2019; Mason & Miller, 2006; Sherman et al., 2019; Üstün & İnan, 2018; Wagaman et al., 2019; Werkmeister Rozas & Garran, 2016; White-Davis et al., 2018). This means that the programs’ theory base was set on the premise that it is supposed to teach its participants something that should be core to their knowledge in their profession. Another theory used by a single study is the idea of increasing the effectiveness of work (Crawford et al., 2017). This also implies that the behaviour, attitudes and skills the program is teaching are integral to the participants’ profession. Some studies focused on actual collaboration and creation of change, either in the communities where the participants work or in collaboration with other healthcare professionals (Bakshi et al., 2015; Flatt-Fultz & Phillips, 2012; Gonzalez et al., 2015; Lax et al., 2019; Tucker et al., 2020).

Eight other studies operated on the premise that healthcare professionals are harming their patients with a lack of skills, thus deepening discrimination (DeLashmutt & Rankin, 2005; Potts et al., 2022; Knaak, 2018; Li et al., 2015; Nelson et al., 2015; O Carroll & O’Reilly, 2019; Üstün & İnan, 2018; Zäske et al., 2014). Furthermore, some of the interventions are based on the theory that stigma is implicit in all structures of healthcare, and therefore simple programs are no longer enough to impact change, there is a need for healthcare professionals to actively fight against organizational stigma (Allen et al., 2013; Geibel et al., 2017; İnan et al., 2019; McAllister, 2008; Sukhera et al., 2020; Wu et al., 2019).

#### Content

More programs focused on individual discrimination combined with some aspects of structural discrimination rather than solely on structural discrimination. Those which focused on structural stigma often centred around social determinants of health, community health, advocacy, and political action while targeting the idea of the responsibility of the professional (Bakshi et al., 2015; Boutain, 2008; Crawford et al., 2017; Dharamsi et al., 2010; Gonzalez et al., 2015; Lax et al., 2019). The studies which focused on individual stigma emphasized more needs-based approaches, which were embedded in communication with patients, and targeted individual healthcare professionals’ stigmatizing beliefs (Ezedinachi et al., 2002; Flatt-Fultz & Phillips, 2012; Knaak, 2018; Li et al., 2015; Potts et al., 2022; Zäske et al., 2014).

Frequently mentioned concepts were those of social justice knowledge, action, and integration as well as health and human rights (Allen et al., 2013; Bakshi et al., 2015; Boutain, 2008; Crawford et al., 2017; Ezedinachi et al., 2002; Jones & Smith, 2014; McAllister, 2008; Webb & Sergison, 2003). These were operationalized within the limits of the participants’ current or future profession. Specific concepts often mentioned were social determinants of health and discrimination, marginalization of certain groups (Allen et al., 2013; Crawford et al., 2017; DallaPiazza et al., 2018; Dharamsi et al., 2010; Gonzalez et al., 2015; Knaak, 2018; Lax et al., 2019; O’Carroll & O’Reilly, 2019). A small proportion of studies focused on stigma specifically, for example, stigma of a specific health condition or racism (Ezedinachi et al., 2002; Fisher-Borne, 2009; İnan et al., 2019; Knaak, 2018; Potts et al., 2022; Sherman et al., 2019; Webb & Sergison, 2003; Werkmeister Rozas & Garran, 2016; Zäske et al., 2014). Some studies focused on the participants’ own biases and actively examining them (Gonzalez et al., 2015; Knaak, 2018; Nelson et al., 2015; Potts et al., 2022; Sukhera et al., 2020; Üstün & İnan, 2018; Wagaman et al., 2019; Wu et al., 2019). Three studies also focused on discrimination from the perspective of the patient (Flatt-Fultz & Phillips, 2012; Üstün & İnan, 2018; Zäske et al., 2014).

**Figure 2.**
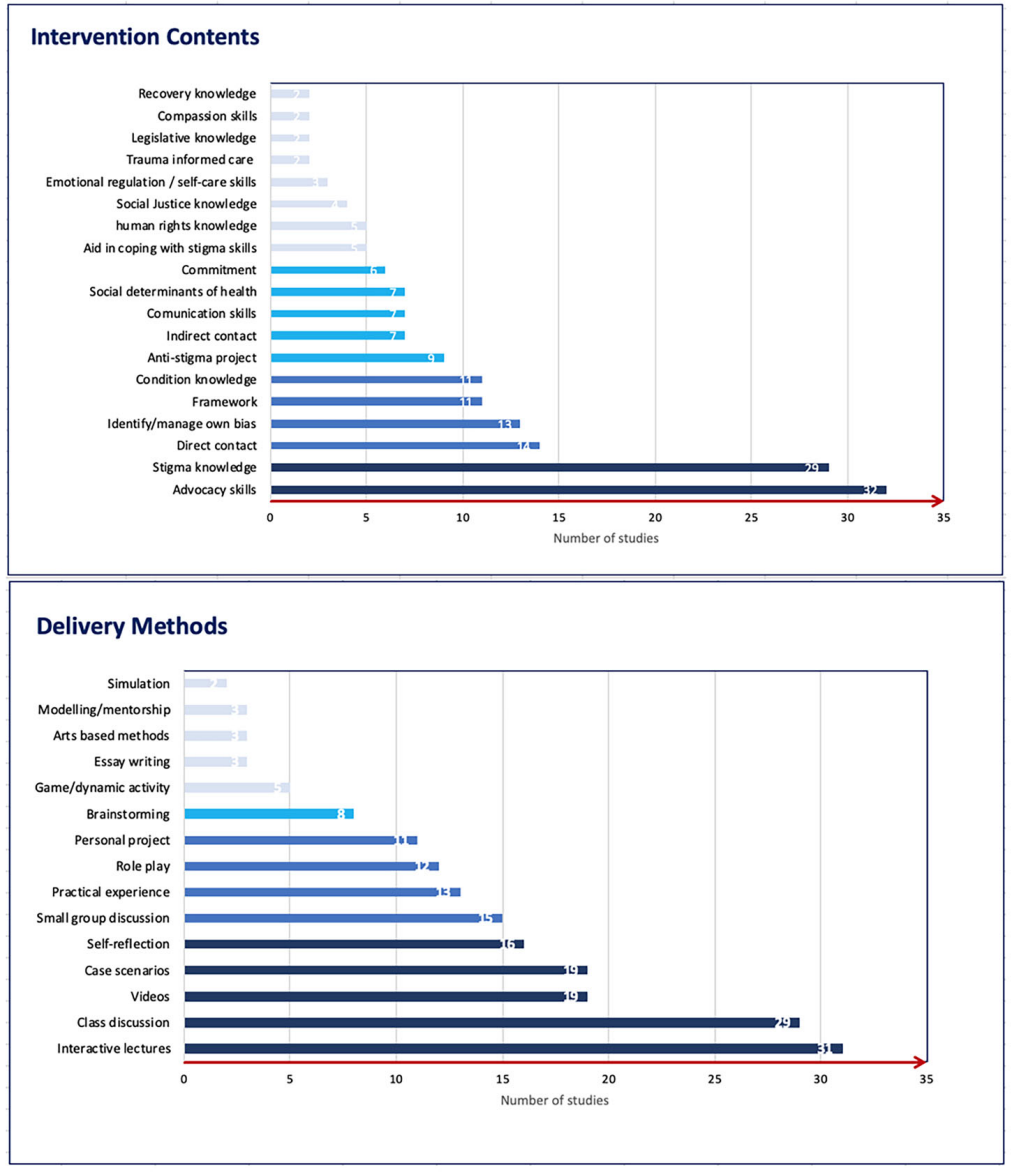
Intervention contents and delivery methods frequencies.

Finally, a large majority of studies focused on teaching correct practices and skills (Allen et al., 2013; Dharamsi et al., 2010; Fisher-Borne, 2009; Geibel et al., 2017; Gonzalez et al., 2015; Griffith & Kohrt, 2016; İnan et al., 2019; Knaak, 2018; Lax et al., 2019; Li et al., 2013, 2015; McAllister, 2008; Sukhera et al., 2020; Üstün & İnan, 2018; Webb & Sergison, 2003; Wu et al., 2019). These can be further categorized as communication skills, the ability to address discrimination in different settings, comfort around topics of stigma, and specific strategies such as defining and working with legislation regarding poverty, human rights activism, screening and referral to appropriate services to mitigate social determinants of health and actively developing projects that address discrimination. Frequencies can be seen in Figure 2 below.

#### Delivery methods

The length of the programs varied, from hours to days or weeks. Most studies included a component of knowledge transmission (Allen et al., 2013; Bakshi et al., 2015; Boutain, 2008; DeLashmutt & Rankin, 2005; Dharamsi et al., 2010; Ezedinachi et al., 2002; Geibel et al., 2017; Gonzalez et al., 2015; İnan et al., 2019; Jindal et al., 2022; Knaak, 2018; Lax et al., 2019; Li et al., 2015; Mason & Miller, 2006; McAllister, 2008; Potts et al., 2022; Sherman et al., 2019; Sukhera et al., 2020; Tucker et al., 2020; Üstün & İnan, 2018; Uys et al., 2009; Wagaman et al., 2019; Webb & Sergison, 2003; Werkmeister Rozas & Garran, 2016; White-Davis et al., 2018; Wu et al., 2019; Zäske et al., 2014). The latter was achieved in several different ways, for example, lecture components on basic facts of discrimination, didactic teaching, a transformative learning approach, roleplays, seminars, workshops, videos, case-based examples. However, some also included more introspective methods of learning such as considering one’s own behaviour and skills through journaling or applying the knowledge learned to own experiences from practice (Bakshi et al., 2015; Boutain, 2008; Crawford et al., 2017; Knaak, 2018). Applying knowledge in practice was mentioned by a proportion of studies. This most often took the form of working on a project alongside local communities, working with mentors, or training other students in a train-the-trainer approach (Bakshi et al., 2015; Dharamsi et al., 2010; Ezedinachi et al., 2002; Üstün & İnan, 2018). Some studies also included learning from people with lived experience through discussions (Lax et al., 2019; Potts et al., 2022; Zäske et al., 2014). Finally, two studies used an online platform or remote video projection to deliver the program (Flatt-Fultz & Phillips, 2012; Knaak, 2018). Frequencies can be seen in Figure 2 below.

### Research question 2 – What is the evidence for the effectiveness and feasibility of the programs?

The results for research question 2 were extracted from 19 studies out of the 41 included and can be seen in Table 2 below. In general, for all the studies some positive, significant outcomes are reported. About half of the studies show changes in mean scores for measures of attitudes (Ezedinachi et al., 2002; Flatt-Fultz & Phillips, 2012; Geibel et al., 2017; Gonzalez et al., 2015; İnan et al., 2019; Knaak, 2018; Lax et al., 2019; Li et al., 2015; Potts et al., 2022; Tucker et al., 2020; Zäske et al., 2014). Ten studies show changes in knowledge (Crawford et al., 2017; Fisher-Borne, 2009; Flatt-Fultz & Phillips, 2012; Geibel et al., 2017; Gonzalez et al., 2015; Knaak, 2018; Li et al., 2015; Nelson et al., 2015; Tucker et al., 2020; Zäske et al., 2014), while only seven show changes in skills (Crawford et al., 2017; Ezedinachi et al., 2002; Lax et al., 2019; Nelson et al., 2015; Wu et al., 2019; Tucker et al., 2020; Zäske et al., 2014).

However, there is great variability in the scales chosen to assess different components of the programs presented in the studies. Furthermore, not all studies assessed knowledge, attitudes, and skills but rather selected some of these components or specific subsets within these components. Therefore, it is difficult to compare the effectiveness of the studies against each other. Finally, it must be noted that the scales chosen to assess discrimination mostly assessed intended behaviour rather than actual completed actions. Finally, two studies chose to assess the impact on patients by directly measuring patient outcomes (Geibel et al., 2017; Uys et al., 2009). Detailed efficacy outcomes can be seen in Table S4 in supplementary materials.

Feasibility or acceptability was measured only in six studies, the specific outcomes can be seen in Table 3 below. Most of the studies report positive outcomes with participants rating the interventions agreeable and acceptable for their practice.

#### Studies included but not processed for data extraction

There are three studies which were not included in the data extraction stage either because they do not fit the inclusion criteria or because they are protocols. It is important to describe these studies to map possible outcomes in future literature. Firstly, Grandón et al. (2019) describe in their protocol an educational program for healthcare professionals which will focus on educational outcomes and contact with people with lived experience to reduce stigma, as well as promoting inclusive behaviours towards people with severe mental disorders. Secondly, Chenneville, Gabbidon and Drake’s (2019) study describes a program delivered by community healthcare workers to youth living with HIV to destigmatize and educate them about the condition. Through qualitative research Chenneville et al., (2019) found that community healthcare workers delivering the program saw a destigmatizing effect on themselves through teaching the program. This finding would tie into other studies mentioned which also utilize a train-the-trainers approach (Ezedinachi et al., 2002). Finally, Maranzan (2016) describes a new learning environment called interprofessional education in which participants learn from other professionals within their field who have experience within the chosen topic. This type of learning could be well suited to learn about and integrate stigma reduction strategies through an open unbiased discussion amongst healthcare professionals.

## Discussion

The current study aimed to assess the existing evidence regarding programs targeted at healthcare professionals which aim to not only destigmatize but also to teach healthcare professionals how to address stigma. The search identified 41 studies from which data was extracted. While the programs differed in many aspects, there were some clear themes within each category explored – theory base, content, and delivery methods. In terms of effectiveness, feasibility, and evaluation there are some concerns regarding the methods employed by each study.

The studies included can be considered of varying quality, with some of them being of very low quality. The quality of the studies included in turn impacts the quality of the current study findings. However, because we focused on program content, no studies which scored low in their quality appraisal were excluded, to allow the extraction of such data. We also included papers which did not include program evaluation but instead aimed to describe the programs in detail and explain the rationale for their creation.

### Feasibility and evaluation

In most papers, feasibility and evaluation were described as practical approaches to the implementation of interventions. Thus, there is a distinct lack of information on barriers to delivery, or resources needed to implement an intervention. Therefore, we describe feasibility and evaluation as concrete aspects of interventions which allow its implementation.

Firstly, in terms of the feasibility of the programs, it is clear that programs can be delivered to either undergraduates or postgraduate trainees, and there were no clear differences in length or delivery formats between courses for these groups. However, only a small sub-set of programs addressed fully qualified professionals, suggesting that it is harder to reach this group.

Secondly, interventions for postgraduate and fully qualified professionals were found largely in certain specialties such as primary care, where they are tailored to participants’ professional roles. This suggests that some specialties may not yet be widely incorporating health advocacy training even where this has been recommended at the national level (Leveridge et al., 2007).

Thirdly, as most of the programs were carried out in high-income countries, the feasibility of such programs in low- and middle-income countries is hard to establish on the basis of the evidence presented. This is also because, as already stated, it is unclear what resources and investments were necessary for the presented interventions in the first place. Furthermore, in different cultures, the needs of patients but also necessary resources may be different, and they may experience stigma in different ways from participants in high-income countries.

In terms of evaluation, very few studies included a pilot stage or a situational analysis to adapt the programs to their contexts. While most studies used a pre-post data collection method, only one conducted a follow-up assessment beyond six weeks. This means that no data on actual behavioural change were collected. Furthermore, as already mentioned no data from patients were collected so the intended impact of the programs could never be directly measured. The findings seem to point to positive outcomes, however, the outcomes that were measured were very variable and therefore it is hard to compare which program was, in fact, more effective. Another factor that complicates the evaluation of the effectiveness of the included studies is that most of them did not effectively link the theory basis or content of the programs to the outcome measures. Therefore, we can only assume implicit theories, such as active learning style may help with knowledge retention regarding discrimination. The above issues could be remedied by a longer follow-up period which would measure actual behavioural changes as well as impacts as perceived by their patients.

### Theory base, content and delivery methods

The theory base for most studies could be summarized intro three main foci– responsibility as part of the professional role, correction of wrongful practices, and collaboration with local communities. However, based on previous literature it seems that embedding the program into morals tied to the role of the healthcare professional seems to lead to better long-term changes. Research has suggested that continued professional development for healthcare professions seems to be more effective if embedded into the concept of social accountability and the professional role (Fleet et al., 2008).

With regard to the content of the programs, there seems to be a split between focusing on broad topics such as social determinants of health versus stigma towards a particular health condition. Broad concepts such as the social determinants of health can be limiting, as they are harder to define or operationalize for training purposes. However, such concepts also invite a much larger conversation which can lead to programs which address the societal structures of stigma. On the contrary, programs which focus on specific mental health disorders invite a more in-depth analysis of the needs of patients with such disorders. They remain tied to a smaller community and may be preferable for more specialized healthcare professionals. Nevertheless, either of these approaches allow the introduction of the spectrum of social accountability and the “fit” between a particular issue and the level (micro, meso, or macro) at which it would best be addressed (Bernard et al., 2019).

Few studies focus on addressing the participants’ biases and stigma directly, rather they educate without assessing the initial levels of discrimination participants may hold. However, realizing one’s own bias may consist of an education outcome in and of itself (Menatti et al., 2012). Further, while all the studies provide their participants with some methods on how to combat stigma, not as many provide specific methods and skills. This is especially important when needing to address structural stigma as this can be a challenge for which healthcare professionals are not prepared. Finally, few studies chose to mention patient perspectives, be that in designing the program or in its content. Very few papers focus on the concept of self-stigma with only a few interventions implementing components of empowerment of people with lived experience. This is a gap in the literature that further ties to the lack of patient perspectives in interventions for the healthcare professional. However, when it comes to experiences of stigma people with lived experience should be considered the experts. Moreover, adapting the program to the needs of the patients would increase its effectiveness. For example, community-based participatory research has often been shown to be an effective way to create programs which are suited to the needs of vulnerable communities (Stacciarini et al., 2011). Furthermore, the creation of a program which does not include people with lived experience input could be considered stigmatizing in and of itself. Without this input, programs which teach healthcare professionals to combat the stigma which their patients experience could easily become paternalistic in their rhetoric.

Regarding delivery methods, while all programs used active learning as a key component, the way in which this was delivered differed. Several studies also lead to the application of the knowledge learned in projects or real-life settings mediated for example via roleplays. Previous research on microaggressions in the classroom setting suggests “the advantage of discussion” is the opportunity for “the ambiguous nature of microaggressions be elucidated for those that may not be aware that bias has occurred” (Boysen, 2012). This is especially useful as subtle forms of stigma are common and many times unnoticed in health professionals (e.g. over protectionist behaviour, lower educational expectations) (Mason & Miller, 2006).

Perhaps most interestingly, some programs required students to work with communities and create long-lasting projects. This meant that not only did the program train the participants but also contributed actively towards destigmatisation.

### Strengths and limitations

A strength of this study is its breadth of coverage, due to the range of databases used and the inclusion of undergraduate and postgraduate trainees and fully qualified professionals across the health professions. However, the review is nonetheless vulnerable to publication bias. The studies included describe programs with positive outcomes, it is very likely that programs or versions of the programs included which did not work were not published. The current study also did not use meta-analysis to analyse the data. This is primarily because of the differences between study designs, populations assessed, and programs. However, the chosen narrative synthesis allows for a robust analysis of the data. Another limitation of this study is the fact that the stages of full-text screening and data extraction were done by only one reviewer for most papers due to the division of the data set after 10% of each half was discussed among co-reviewers.

## Conclusions

The reason for undertaking the current study was to inform the development of a program to focus on mental health stigma and how mental health professionals can reduce it. First, the program should be based on theories encompassing both the structural and individual levels. It should contain clear definitions of structural and individual-level stigma and operationalize it using data to show the impacts on patients. Second, it should draw on the idea of the professional role of healthcare providers and their professional accountability. Third, in terms of the inclusion of people with lived experience, there is a need to work alongside vulnerable communities and individuals. For example, Schwartz (2002) outlines that for such cooperation to happen there is a need to have clear guidelines for both the practice and education of healthcare professionals. Regarding some of the programs, there is also a risk that communities may end up feeling exploited. Program participants may gain the impression that projects and collaborations are being done in a sense of optical allyship – meaning that the programs are done in a tokenistic fashion to simply achieve an outcome imposed by current trends or societal pressure. On the other hand, there is a risk of an overly protective stance on the part of professionals, as a result of trying to shield patients from the stigma which reduces their social and economic opportunities. Healthcare professionals’ programs should therefore focus on empowering rather than protecting patients, in both the process of program development and the program content. Moreover, since the impacts of stigma can be seen in social determinants of health, healthcare professionals working with vulnerable populations will need to learn more specific skills in relation to such determinants, such as advocacy for economic stability or educational achievements. Skills such as capacity building may therefore be suitable for such situations.

In order to offer all the above qualities, the studies should improve their research designs and inclusion of people with lived experience. Situational analysis and piloting would ensure that the program is sufficiently tailored to the healthcare professionals’ setting as well as their patients’ context. Secondly, there is a need to identify a theory to achieve modelling of how the program and works to achieve its intended outcomes. Thirdly, the program should be tailored to the participants’ context which means assessing the feasibility, acceptability, sustainability, and perceived effectiveness of the program. Studies should aim to use rigorous study designs including longer follow-up periods (Craig et al., 2008), and assess skills rather than intended behaviour. Lastly, studies should try to include measures of the direct impact on patients.

## Supplementary Material

Supplemental Material

## Data Availability

Data sharing is not applicable to this article as no new data were created or analyzed in this study.
