# Supplementary material for: Anti-stigma advocacy for health professionals: a systematic review
Source: J Ment Health. 2023 Mar 15;33(3):394–414. doi: 10.1080/09638237.2023.2182421 (PMC10173949; doi:10.1080/09638237.2023.2182421)
Supplement: Supplemental Material [file IJMH_A_2182421_SM6896.docx]

Supplementary material

Table S1: PICOs for both research questions

| **PICO research question 1**    **What are the theory base, content and delivery methods of the programs?** | **PICO research question 2**    **What is the evidence for the effectiveness of the programs?** |
| --- | --- |
| P - health professionals | P - health professionals |
| I - interventions/programs to reduce patient’s discrimination and health impacts (defined in terms of theory base, content and delivery methods) | I - empirical studies examining the effect of interventions/programs on the reduction of patient’s discrimination and health impacts |
| C - no control - pre and post measurements or just post measurements, controls - health professionals who have not been trained, existing/best available training | C - controls - health professionals who have not been trained, pre and post measurements, existing/best available training |
| O - impact on healthcare professional defined as    - Knowledge on discrimination and its health impact  - Attitudes to people with the characteristic/health condition covered by the training  - Attitudes to addressing discrimination as part of one’s professional role  - Confidence in personal ability to address discrimination  - Skills in addressing discrimination as part of one’s professional role, through education, challenging, lobbying for improved services, or other means.  - Behavioural outcomes- actions taken to address discrimination | O - impact on discrimination and health outcomes of patients through knowledge, skills and attitudes of trained health professionals |

Table S2: Search terms

| **Health professional terms** | **Stigma terms** | **Intervention terms** |
| --- | --- | --- |
| (Health care professional*) OR physician*1.tw or (Health* adj2 personnel*) OR (Health* adj2 student*) OR Doctor*1 OR Psychiat* OR (clinical psych*) OR nurs*.tw OR midwi*.tw OR (occupational therap*).tw OR resident or  physiotherapy*.tw OR (medical student*1).tw OR (health* worker*1).tw OR Pharmacist*1.tw OR (Pharmacy student*1).tw OR Paramedic*.tw OR (ambulance service).tw OR dietitian*1.tw OR dietician*1.tw OR (mental health social worker*1).tw OR  (social worker*1).tw OR Dentist*1.tw OR Surgeon*1.tw OR Medic*1.tw OR Clinician*1.tw OR Physician*1.tw OR (case manager*1).tw OR (key worker*1).tw OR (keyworker*1).tw OR (keyworker*1).tw OR psychotherapist*1.tw OR hospital*.tw OR a&e.tw OR (Emergency department*1).tw OR (Health* provider*1).tw OR (Health* staff).tw OR Obstetrician*1.tw OR (Dental hygienist*1).tw OR (general practitioner*1).tw OR Gynaecologist*1.tw OR (Rehabilitation worker*1).tw OR (Vocational rehabilitat*).tw OR (Work rehabilitat*).tw OR (Occupational rehabilitat*).tw OR (direct support professional*1).tw or (Family medicine doctor*1).tw OR (Family medicine specialist*1).tw | (((patient* adj advocac*) or (health adj advocac*) or microaggression* or (social justice) or advocacy or (social adj accountabilit*) or (social adj2 responsibilit*) or (implicit bias) or (stereotyp* adj2 attitude*1) or stereotyp* or discriminat* or ignoran* or (human rights abuse*1) or (human rights) or misconception*1 or empowerment or misperception*1 or prejudi* or (less adj access) or (under adj treatment) or racist behavio?r*1 or (abusive adj behavior?r*1) or patroniz* or patronis* or no access or (racist adj attitud*) or (non adj treatment) or racism or stigma* or (diagnostic adj overshadow*) or misdiagnos* or judge* or (negative adj2 attitud*) or margin* or hostil* or disrespect* or (negative adj opinion*1) or (negative adj view*1) or (social adj justice) or (negative adj image*) or (Health adj status adj disparitie*1) or disparirie*1 or (Rejecting adj behavio?r*1) or injustice* or (social adj (distanc* or rejection or exclusion)) OR (Cultural competenc*) or (Culturally competent care) | (intervention* or (stigma* adj2 intervent*) or (antistigma* adj2 intervent*) or trainning* or training or skill* or competenc* or program* or knowledge*1 or attitud* change or teaching or learning or instruction* or course or retraining or curricul* or (Educat* adj2 intervent*) or Educat*) |

MeSH terms:

| **Database** | **Stigma** | **Intervention** | **Health Professional** |
| --- | --- | --- | --- |
| **PsychINFO** | health personnel/ or medical students/ or Nursing students/ or Therapists/ | Clinical methods training/ or Medical education/ or Education/ or Curriculum/ | Prejudice/ or Social discrimination/ or Stigma/ or Student attitudes/ or health personnel attitudes/ |
| **Medline** | Prejudice/ OR Social discrimination/ OR Social Responsibility/ OR  Stereotyping/ OR Social stigma/ OR Patient advocacy/ OR Social distance/ OR Rejection psychology/ OR racism/ | Exp Training support/ OR Exp Simulation training/ OR Exp Health personnel, education/ OR Exp Teacher training/ OR Exp Education, professional/ OR Exp Education, medical/ or curriculum/ or program evaluation/ or teaching/ | Exp Health personnel/ OR Exp Students, health occupations/ OR exp health occupations/ OR Exp Social workers/ OR Exp Nurses/ OR Exp Midwifery/ OR Exp General practitioners/ OR Exp physicians, family/ OR  Exp Community mental health services/ OR “Attitude of Health Personnel”/ or physicians/ |
| **EMBASE** | Prejudice/ OR Social discrimination/ OR Social Responsibility/ OR Stereotyping/ OR Social stigma/ OR Patient advocacy/ OR Social distance/ | Simulation training/ OR medical education/ OR Nursing education/ OR *Education/ | Health care personnel/ OR Health Students/ OR Social worker/ OR Nurse/ OR  Midwife/ OR General practitioner/ |

Table S3: ICROMS scores

| **ICROMS SCALE** | | | | |
| --- | --- | --- | --- | --- |
| **Author** | **Design** | Total | % | Inclusion |
|  |  |  |  |  |
|  |  |  |  |  |
| Burdett et al. [ | Cross-sectional | 14 | 46.7% | 0 |
| DallaPiazza et al. | Cross-sectional | 20 | 66.7% | 0 |
| Sherman et al. | Cross-sectional | 19 | 63.3% | 0 |
| Webb | Cross-sectional | 19 | 63.3% | 0 |
| Bakshi et al. | Qualitative | 18 | 69.2% | 1 |
| Dharamsi et al. | Qualitative | 17 | 65.38% | 1 |
| Gonzalez et al. | Qualitative | 15 | 57.7% | 0 |
| Jindal et al. | Qualitative | 15 | 57.7% | 0 |
| Allen et al. | UPPI | 21 | 70% | 0 |
| Crawford et al. | UPPI | 23 | 76.7% | 1 |
| DeLashmutt & Rankin | UPPI | 16 | 53.3% | 0 |
| Fisher-Borne | UPPI | 25 | 83.3% | 1 |
| Geibel et al. | UPPI | 23 | 76.7% | 1 |
| Gonzalez et al. | UPPI | 20 | 66.7% | 0 |
| Knaak | UPPI | 20 | 66.7% | 0 |
| Lax et al. | UPPI | 12 | 40% | 0 |
| Nelson et al. | UPPI | 16 | 53.3% | 0 |
| İnan et al. | UPPI | 24 | 80% | 1 |
| Sukhera et al. | UPPI | 17 | 56.7% | 0 |
| Uys et al. | UPPI | 22 | 73.3% | 1 |
| White-Davis et al. | UPPI | 24 | 80% | 1 |
| Wu et al. | UPPI | 19 | 63.3% | 0 |
| Tucker et al. | UPPI | 15 | 57.7% | 0 |
| Shah et al. | CPPI | 24 | 85.7% | 1 |
| Potts et al. | CPPI | 25 | 89.3% | 1 |
| Ezedinachi et al. | RCT | 26 | 81.25% | 1 |
| Li et al. | RCT | 28 | 87.5 | 1 |
| Li et al. | RCT | 26 | 81.25% | 1 |
| Zäske et al. | RCT | 22 | 68.7% | 1 |
| Boutain | Descriptive | / | / | Cannot assess |
| O Carroll & O’Reilly | Descriptive | / | / | Cannot assess |
| Fisher et al. | Descriptive | / | / | Cannot assess |
| Flatt-Fultz & Phillips | Descriptive | / | / | Cannot assess |
| Griffith & Kohrt | Descriptive | / | / | Cannot assess |
| Jones & Smith | Descriptive | / | / | Cannot assess |
| Mason & Miller | Descriptive | / | / | Cannot assess |
| McAllister | Descriptive | / | / | Cannot assess |
| Sheely-Moore & Kooyman | Descriptive | / | / | Cannot assess |
| Üstün & İnan | Descriptive | / | / | Cannot assess |
| Wagaman et al. | Descriptive | / | / | Cannot assess |
| Werkmeister Rozas & Garran | Descriptive | / | / | Cannot assess |
| *Abreviations*: %: Percentage of total item score, UPPI: uncontrolled pre-post intervention, CS: cross-sectional, CPPI: controlled pre-post intervention, RCT: randomized controlled study | | | | |

| **Author**  Table S4: Detailed efficacy outcomes  **Year Country** | **Design** | **Sample size** | **Sampling** | **Outcome** | **Measurement** | **Follow-up** | **Results** | **Limitations** | **Response rate** |  |
| --- | --- | --- | --- | --- | --- | --- | --- | --- | --- | --- |
| **Allen et al.**  (2013)  Australia | UPPI | 33 | Convenience sample (mandatory academic activity) | Attitude, knowledge | Quick discrimination index, Transcultural self-efficacy tool (confidence in practicing cross-cultural nursing). | Post-intervention | No improvement in attitude scale. Improvement in transcultural self-efficacy tool (only in cognitive subscale) | Low response rate, no control group | 13.2% |  |
| **Crawford et al.**  (2017)  Australia | UPPI | 120 | Convenience sample | Knowledge, skills | Students were asked to list all the human rights they knew in the pre- and post-questionnaires;  The Modified Human Rights Scale (mHRS) | Post-intervention | Knowledge improved, no statistical significance studied. (4.55 (SD=2.03) to 7.7 (SD=2.94) human rights listed and 40.30 (SD = 11.87) to 89.48 (SD = 9.09) knowledge in general).  Skills improved, no statistical significance studied. (52.58 (SD = 12.48) to 73.67 (SD = 14.96) in confidence regarding working towards human rights) | No quantitative analysis, short follow-up. | 48.3% |  |
| **Ezedinachi et al.**  (2002)  Nigeria | RCT | 1552 | Cluster randomization of hospitals (38) in two states. According to staff facilities to ensure representation of different hospital levels. | Skills, attitude | Bespoke scale (created based on previous focus group) | Post-intervention | **Skills:** Significant increase in perceived HIV-related clinical skills. Specifically, in judging skills in assessing psychosocial complications associated with HIV=AIDS (B: -0.1, p=0.02,); ability to provide care to AIDS patients (B= -0.096, p=0.02);  **Attitudes:** Less fear (B= -0.156, p=0.00) and more sympathy and responsibility (B= -0.329, p=0.00) | Dificulty in matching control and intervention sites (although baseline characteristics were entered as covariate in analysis), incomplete follow-up, possible ceiling effect. | Not reported |  |
| **Author Year Country** | **Design** | **Sample size** | **Sampling** | **Outcome** | **Measurement** | **Follow-up** | **Results** | **Limitations** | **Response rate** |  |
| **Fisher-borne** **et al.**  (2009)  US | UPPI | 54 | Convenience sample (mandatory activity) | Knowledge, attitudes and skills. | Likert survey measuring knowledge of LGBT and stigma, attitudes towards LGBT people and ability to deliver care for LGBT people (confidence in interviewing and ability to address negative stereotypes). | Post-intervention, 3 months | Knowledge improved significantly post-intervention and maintained 3 months after. There was no change in attitude or skills subscale. | Small sample size, not validated self-report, measurement, short follow-up, possible ceiling effect of skills and attitudes | 95% |  |
| **Geibel et al.**  (2016)  Bangladesh | UPPI | 300 | Convenience sampling | Attitude, knowledge,  behaviour, service user satisfaction. | Likert survey measuring provider attitudes toward PLWA.  Likert survey measuring probability of providing care for a PLWA.  Likert survey measuring knowledge on policies protecting PHLIV from discrimination.  Cross-sectional interview measuring client satisfaction with overall services. | Post-intervention and 6 months | Most attitudes improved post-intervention significantly. Some after initial training (fear of disease transmission from specific population), while others (decrease in unwilling to provide services) only after the 2º training. Few had no change (agree HIV is a punishment).  Post-intervention providers were significantly more likely to report having provided care for PLWH. P  Providers knowledge on health facility policies protecting PLHIV from discrimination increased significantly.  Clients were more likely to report discussing stigma with providers after the 2º training. Enacted stigma was rarely reported after the first training but still significantly decreased to 0% after the 2ª training. Clients who reported being dissatisfied with overall quality of service decreased significantly after the first training and further more after the 2º. | No control group, risk of desirability bias in health provider outcomes, mostly female participants (limiting generalizability), client outcome based on probability sample. | 75% |  |
| **Author Year Country** | **Design** | **Sample size** | **Sampling** | **Outcome** | **Measurement** | **Follow-up** | **Results** | **Limitations** | **Response rate** |  |
| **Gonzalez et al.**  (2015)  US | UPPI | 48 | Convenience sample, self-selected. | Knowledge, attitude, self-confidence | Bespoke measure | Post-intervention | The knowledge, attitudes, and self-reported confidence domains all showed a statistically significant increase. Each individual knowledge question, except one about physicians’ assumptions, showed a statistically significant increase | Possible selection bias, lack comparison group, behaviour change was not assessed, | 81.25% |  |
| **Knaak et al.**  (2018)  Canada | UPPI | 232 | Convenience sample | Attitude | OMS-HC | Post-intervention, 3 months | OMS-HC improved significantly. 22.6% relative improvement from pre to post training, effect size (Cohen’s d)= 1.04, p<0.001.  Minimun detectable change method showed (with 90% confidence) only 0.6% of the sample became more stigmatizing post-intervention.  Follow-up at three months showed a loss of gains from the time of course completion with no significant diference from baseline. | Limited effect over time (need for booster sessions). | 77.16% at post intervention, 14% at 3 months. |  |
| **Lax et al.** (2019)  US | UPPI | 78 | Convenience sampling, self-selected | Attitude, behaviour | Bespoke measure using 5-point Likert scale about attitudes, comfort and screening practices of social determinants of health (SDH). | Post-intervention | Attitude towards advocacy did not changed.  Perception on how well trained they felt to discuss SDH with patients in the ambulatory increased significantly (p=0.02).  Comfort in discussing SDH with patients ( P = .001.), comfort in advocating for patients’ at the individual level (p = 0.04) and at community level (p=0.016) improved signifcantly.  Self-reported screening practices for income (p=0.025), education (p = 0.008), and legal issues (p = 0.012) improved significantly.  Self-reported engagement in legislative email (p = 0.3); phone (p = 0.94); in person (p = 0.17) showed no differece, but only on social media (p= 0.02.) | Possible information and recall bias (self-report), no control group, short follow-up, objective behaviour change not assessed. | 70% |  |
| **Li et al.**  (2015)  China | RCT | 77 | Randomized cluster sampling | Knowledge, attitude, | Bespoke measure , MICA, MAKS, RIBS | Post-intervention, 6 months, 12 months | Kowledge of mental health increased significantly on intervention group at 6 and 12 months follow up.  Attitude towards mental health improved significantly in all measures, but at different time points post intervention:  **MICA:** Only at 6-month after intervention, mean scores of the intervention group decreased vs the control group (p < 0.01)  **RIBS:** post-training, at 6-month, and at 12-month follow-up mean scores of RIBS of the intervention group increased significantly more vs. the control group (p < 0.01, p < 0.001, p < 0.001)  **MAKS:** At 6-month and 12-month, mean scores of the intervention group increased vs the control group (both p < 0.05) | Small sample size, no measure on patient outcomes. | Not reported |  |
| **Author Year Country** | **Design** | **Sample size** | **Sampling** | **Outcome** | **Measurement** | **Follow-up** | **Results** | **Limitations** | **Response rate** |  |
| **Li et al.**  (2013)  China | RCT | 456 | 40 randomly selected hospitals,  randomized 1:1 to intervention or control.  Convenience sampling of participants. | Behaviour reported by target population and observer. | Survey reporting frequency of intervention message diffusion (reducing HIV stigma and equal treatment) in hospital.  Likert survey on perceived HIV stigma reduction and improvements on equal treatment of patients perceived by observer HCP. | 6 and 12 months | Participant reported message diffusion frequency increased significantly at 6 and 12 months.  Stigma reduction and improvement in equal treatment of patients perceived by observer HCP was significant at 6 and at 12 months vs control. | Self-report (desirability bias) | Not reported |  |
| **Nelson et al**.  (2015)  US | UPPI | 19 | Convenience sampling | Knowledge, skills | 5 statement likert scale measuring awareness of racism, knowledge on the impact of racism and perception on ability to deliver care for patients of colour. | Post-intervention | Awareness level of racism in the US, knowledge on the impact of racism on health care delivery and Individual`s perception on their ability to deliver care for patients of colour increased significantly. | Small sample size | Not reported |  |
| **Potts et al.**  (2022)  UK | CPPI | 570 | Convenience | Knowledge, attitudes, skills | The mental health knowledge scale (MAKS),  The mental illness clinicians’ attitudes scale (MICA2), Objective Structured Clinical Examination (OSCE),  Jefferson Scale of Patient Perception of Physician Empathy (JSPPPE) | Pre and Post-intervention | Significant improvement in attitudes, knowledge and skills vs controls. | Missing data across study sites, variable sampling methods across study sites | Not reported |  |
| **Inan et al.** (2018) Turkey | UPPI | 68 | Convenience sample | Attitude | Social Distance Scale (SDS). | Post-intervention | SDS improved significantly. | No control group, small sample size, applied the same scales multiple times in a short period. | 94% |  |
| **Author Year Country** | **Design** | **Sample size** | **Sampling** | **Outcome** | **Measurement** | **Follow-up** | **Results** | **Limitations** | **Response rate** |  |
| **Shah et al.** (2014) India | CPPI | 91 | Convenience sampling (mandatory academic activity) | Attitude, Behaviour. | Rated agreement with coercive policies.  Multiple choice questions measuring intent to discriminate during two hypothetical interactions with PLWA. | Post-intervention | Intent to discriminate when dispensing medication improved significantly vs controls, but not intention to discriminate when drawing blood.  There was no significant decrease in the number of coercive policies endorsed. | Small sample size, convenience sampling, short follow-up. | 100% |  |
| **Sukhera et al.**  (2019)  Canada | UPPI | 69 | Convenience sample. | Attitude | Mental illness Clinician Attitudes scale version 4 (ICA v4), Brief Mental Illness Attitudes scale (BMIAS) | Post-intervention, 6 months. | -In Pediatric Emergency BMIAS decreased significantly after intervention (with moderate mean difference (0.432), but not at 6 months follow-up. MICAv4 showed no significant change.  -In Pediatric Inpatient, MICAv4 significantly decreased with a large effect size post-intervention but not at 6 months follow-up. BMIAS scale showed no significant change.  -In the Adult Emergency Department there was no significant change on MICAv4 or BMIAS. | No control group, small sample size, underrepresentation of Adult Emergency Department professionals. BMIAS scale not widely used. | Not reported |  |
| **Tucker et al.**  (2020)  USA | CPPI | 121 | Convenience sampling | Attitudes, beliefs, behavioural intentions | Day’s Mental Illness Stigma Scale-Anxiety (DMISS), Medical Condition Regard Scale (MCRS), Opinions about Mental Illness- Interpersonal Etiology (OMI), Attitudes Toward Psychiatry, Doctors’ Attitudes Toward Collaborative Care- Psychiatric Consultation (DACC-MH), Integration of Counseling Skills, Competent Caring Questionnaire (CCQ) | 3 months | Attitudes: lower levels of anxiety and lower desires for social distance at post-test and at 3 months.  Beliefs: improved beliefs at post-test, and at 3-month follow-up.  Behaviour: improved on a vignette-based measure of behaviour (i.e., Competent Caring Questionnaire) at post-test and at 3- month follow-up.  Willingness to integrate counseling into routine medical care at post-test, was not maintained at 3-month follow-up. | Possible participant bias | 52% |  |
| **Uys et al.**  (2009) Lesotho, Malawi, South Africa, Swaziland and Tanzania | UPPI | 84 | Convenience sampling | Target population:  Attitude,  Stigmatized population: Self-efficacy, Self-stem, perceived stigma. | HIV=AIDS Stigma Instrument–Nurse and HIV=AIDS Stigma Instrument–PLWA. General Self-Efficacy Scale. Self-Esteem Scale. | 1 month | No change in stigmatizing attitudes of nurses.  PLWA showed a decrease in overall perceived stigma, and increased self-stem after intervention. There was no change in self-efficacy. | Small sample size, short follow-up, possible selection bias. | Not reported |  |
| **Author Year Country** | **Design** | **Sample size** | **Sampling** | **Outcome** | **Measurement** | **Follow-up** | **Results** | **Limitations** | **Response rate** |  |
| **White-Davis et al.**  (2018)  US | UPPI | 80 | Self-selected convenience sample | Knowledge, attitudes, skills, self-confidence | Likert survey measuring knowledge of the impact of racism on health inequities, strategies to address racism, commitment to make personal changes in addressing health disparities and self-confidence in ability to make strategies to improve the racial climate. | Post-intervention | Knowledge of racism, strategies to address racism, commitment to make personal changes and confidence in making strategies to improve racial climate significantly improved. | Self-selected sample. No demographic information. Lack of control group. | 90% |  |
| **Wu et al.**  (2019)  US | UPPI | 468 | Convenience sample (mandatory academic activity) | Knowledge, skills. | Likert survey measuring understanding, recognizing, and addressing unconscious bias. | Post-intervention | Confidence in understanding, recognizing and addressing unconscious bias improved significantly. Ability to effectively manage prejudice situations improved significantly. | No demographic information, didn’t measured behavioural outcomes. | 80% |  |
| **Zaske et al.**  (2014) Germany | CPPI | 123 | Self-selected convenience sample. | Attitude, skills, behaviour. | Social Distance Scale (SDS).  Bespoke scale – measuring skills and behaviour in the last 2 months. | Post-intervention, 3 months | -SDS improved significantly vs controls, post-intervention and 3 months after.  -Talking about the importance of stigma in the past 2 months improved significantly with intervention.  -Skills to identify and address stigma significantly improved with intervention. item “acting if one patient is being unfairly treated”, didn`t changed. | Self-assessment, potential selection bias, no follow-up survey in control group, no differentiation per professional group. | Post-intervention 72.2-93.1%  3 months: 48.3% |  |
| *Abreviations*: UPPI: uncontroled pre post intervention, CPPI: controlled pre post intervention, RCT: randomized controled trial, vs: versus, HCP: health care professional, LGBT: lesbians, gays, transsexual and transgender, HIV: human inmunodeficiency virus, AIDS: adquired inmunodeficiency disease, PLWA: people living with AIDS | | | | | | | | | | |
